# Asthma and elevation of anti-citrullinated protein antibodies prior to the onset of rheumatoid arthritis

**DOI:** 10.1186/s13075-019-2035-3

**Published:** 2019-11-21

**Authors:** Alessandra Zaccardelli, Xinyi Liu, Julia A. Ford, Jing Cui, Bing Lu, Su H. Chu, Peter H. Schur, Cameron B. Speyer, Karen H. Costenbader, William H. Robinson, Jeremy Sokolove, Elizabeth W. Karlson, Carlos A. Camargo, Jeffrey A. Sparks

**Affiliations:** 1 Division of Rheumatology, Inflammation, and Immunity, Brigham and Women’s Hospital, 60 Fenwood Road, #6016U, Boston, MA 02115 USA; 2 Harvard Medical School, Boston, MA USA; 3 Stanford University School of Medicine, Palo Alto, CA USA; 4 VA Palo Alto Health Care System, Palo Alto, CA USA; 5 GlaxoSmithKline, Vienna, Austria; 6 Massachusetts General Hospital, Boston, MA USA

**Keywords:** Asthma, Anti-citrullinated protein antibody, Rheumatoid arthritis, Pathogenesis

## Abstract

**Background:**

Anti-citrullinated protein antibodies (ACPA) are central to rheumatoid arthritis (RA) pathogenesis and may develop at inflamed mucosa. We investigated whether asthma, a disease of airway mucosal inflammation, was associated with elevated ACPA before RA diagnosis.

**Methods:**

We performed a nested case-control study among women in two prospective cohorts, the Nurses’ Health Study (NHS; 1976–2014) and NHSII (1989–2015). Blood was obtained on a subset (NHS: 1989–1990; NHSII: 1996–1999). Cases met 1987 ACR or 2010 ACR/EULAR RA criteria by medical record review and were classified as seropositive (ACPA+ or rheumatoid factor positivity) or seronegative by clinical laboratory testing at diagnosis. We identified RA cases with blood drawn before the date of RA diagnosis (index date), matching each to three controls by age, cohort, year, time from blood draw to index date, and menopause. Pre-RA ACPA elevation for cases was defined as >99th percentile of the control distribution on a research assay composed of autoantibodies targeting citrullinated protein epitopes or positivity on the second-generation commercial assay for cyclic citrullinated peptide. Asthma status and covariates were obtained through biennial questionnaires before blood draw. Conditional logistic regression estimated ORs and 95%CIs for RA by pre-RA ACPA and clinical serostatus, adjusted for matching factors, smoking pack-years, passive smoking, and body mass index (BMI).

**Results:**

We identified 284 incident RA cases and 849 matched controls; mean age at the index date was 61.2 years (SD 10.1). Blood was drawn 9.7 years (mean; SD 5.8) before the index date. We identified 96 (33.8%) RA cases with elevated pre-RA ACPA. At blood draw, 17.7% of pre-RA ACPA+ cases and 6.3% of matched controls (*p* = 0.0008) reported clinician-diagnosed asthma. After adjusting for matching factors, smoking pack-years, passive smoking, and BMI, asthma was significantly associated with pre-RA ACPA+ RA (OR 3.57, 95%CI 1.58,8.04). Asthma was not associated with overall RA (OR 1.45, 95%CI 0.91,2.31), but was significantly associated with seropositive RA (OR 1.79, 95%CI 1.01,3.18).

**Conclusions:**

Asthma was strongly associated with ACPA elevation in blood drawn prior to RA diagnosis, independent of smoking. Chronic mucosal airway inflammation may contribute to ACPA development and RA pathogenesis.

## Introduction

The mucosal paradigm for seropositive rheumatoid arthritis (RA) pathogenesis postulates that RA may develop after loss of immune tolerance due to environmental triggers at mucosal surfaces in genetically susceptible individuals [1, 2]. Smoking is an established risk factor for seropositive RA [3, 4]. The biologic effect of smoking or other inhalants (such as cadmium in pollution [5], asbestos [6], silica [7], textile dust [8], coal [9], or diesel fumes [10]) on RA risk may be due to induction of local pulmonary mucosal inflammation at the bronchioles and alveoli, leading to protein citrullination [11] and the aberrant formation of anti-citrullinated protein antibodies (ACPA) in those with the *HLA-DRB1* shared epitope [12–18]. ACPA have been measured in the circulation years prior to clinical RA onset and increase the risk for progression to clinical RA [13, 19–23]. Since many non-smokers develop seropositive RA, other causes of chronic mucosal inflammation may also be important in RA pathogenesis. Asthma is one of the most common chronic diseases, affecting 30 million (10.1% of adults) in the USA [24], and is characterized by chronic mucosal airway inflammation.

Several previous studies have suggested that asthma is associated with increased RA risk [24–32], but none have investigated asthma and ACPA status. Studies using administrative datasets reported that patients with asthma had an increased RA risk compared to those without asthma, but were unable to adjust for smoking or investigate RA risk by serostatus [24–29]. Case-control studies have also found that asthma may increase RA risk, independent of smoking status [24, 25]. These findings may be limited by recall bias among studies that included prevalent RA, history of smoking of unknown intensity/duration, and lack of data on ACPA/rheumatoid factor (RF) status [31, 32]. Identifying asthma as a risk factor for ACPA development may extend the mucosal paradigm of RA pathogenesis beyond smoking, serving as a link between airway abnormalities and RA risk, particularly for ACPA+ RA.

We performed a nested case-control study of incident RA with matched controls among women from two large prospective cohorts to investigate asthma as a potential risk factor for RA by pre-RA ACPA status. Asthma diagnoses as well as smoking pack-years and other potential confounders were prospectively collected prior to RA onset. We used blood banked years prior to RA diagnosis (index date) in cases and their matched controls to measure ACPA status prior to RA onset. We investigated the association between asthma and RA, overall and defined by RA phenotype (elevation or absence of pre-RA ACPA) at the time of blood draw, then based on serologic status by medical record review time of clinical diagnosis. We hypothesized that asthma would be associated with increased risk for pre-RA ACPA+ RA compared to no asthma, even after adjusting for smoking.

## Methods

### Study design and population

We conducted a nested case-control study among women in the Nurses’ Health Study (NHS) and NHSII, two large prospective cohorts of female registered nurses. The NHS is composed of 121,700 women aged 30–55 at time of enrollment in 1976. Similarly, the NHSII enrolled 116,429 women who aged 25–42 at baseline in 1989. Detailed data on lifestyle, diseases, medications, and family history were obtained on biennial questionnaires during follow-up. All components of this study were approved by the Partners HealthCare Institutional Review Board.

NHS and NHSII participants were asked to donate blood samples that have been stored in aliquots at −70 °C for research use. In the NHS, 27% of women donated blood between 1989 and 1990; in the NHSII, 25% of women donated blood between 1996 and 1999. In this study, we analyzed cases and controls who donated blood and then later developed RA or were chosen as a non-RA matched control. RA cases were diagnosed up to 2014 in the NHS and 2015 in the NHSII.

### RA cases

We previously reported in detail on the methods for RA case identification [33]. Briefly, women who self-reported RA were mailed a supplemental connective tissue screening questionnaire [34]. If positive, medical records were obtained and reviewed by two rheumatologists. All cases met the 1987 ACR (American College of Rheumatology) or 2010 ACR/EULAR (European League Against Rheumatism) RA criteria [35, 36]. Dates of symptom onset and clinical RA diagnosis, as well as clinical laboratory testing around the time of diagnosis, were obtained by medical record review. We defined incident seropositive RA as either cyclic citrullinated peptide (CCP) or RF positivity above the upper limit of normal on the local clinical assay. We defined incident seronegative RA as both CCP and RF negativity. Since CCP was not routinely used clinically until the early 2000s [37], we relied solely on RF testing from medical records to define clinical RA serostatus at time of diagnosis for women who were diagnosed with RA in prior years.

For this analysis, we defined RA cases as women who donated blood and were subsequently diagnosed with RA. The index date was the date of RA diagnosis for cases. To ensure that all assays were performed on blood before RA onset, we required at least 3 months between blood donation and the date of initial RA symptoms.

### Matched controls

For each case, we chose three controls who had never reported RA or other connective tissue diseases as of the index date and had donated blood. We matched each incident RA case to controls by age at blood draw (within 1 year), cohort, time of day in blood collection, fasting status, menopausal status, and postmenopausal hormone use.

Blood from each unit (one case and three controls) without labelling identifying case/control status were sent together for laboratory assays for testing.

### Measurement of ACPA

We used two separate laboratory assays to test for ACPA: a research multiplex assay and a commercial CCP2 assay.

#### Research multiplex ACPA assay

The research ACPA test was performed at Stanford University (Palo Alto, CA, USA) using a bead-based multiplex assay that has previously been described in detail [38–41]. Briefly, synovium-specific citrullinated and non-citrullinated protein antigens were conjugated to spectrally distinct beads using the Bio-Plex multiplex assay platform (Bio-Rad Laboratories, Hercules, CA, USA) and were analyzed using the Luminex 200 (Luminex, Austin, TX, USA). Each plate used established samples with no, low, medium, or high reactivity as internal controls.

Plasma from each pre-RA case and matched controls was added to the bead mix, and the reactivity was measured in raw fluorescent intensity units. For these analyses, we only considered antibodies against citrullinated proteins that passed quality control. There were three separate batches sent for testing. All batches tested for citrullinated antibodies against epitopes on the following proteins: apolipoprotein A, apolipoprotein E, biglycan, clusterin, enolase, fibrinogen, fibronectin, filaggrin, histone 2A, histone 2B, and vimentin. There were multiple epitopes targeted for some of these proteins (apolipoprotein E, clusterin, fibrinogen, histone 2A, histone 2B, and vimentin). The composition of each batch of the research ACPA assay varied slightly; a small number of citrullinated antigens were added to the assay while a small number of others were no longer tested. The assay also included a research test for CCP.

Since these research assays did not have a clinical cutpoint for ACPA elevation, we determined ACPA elevation among the pre-RA cases using the control distribution. Elevated ACPA was defined as >99th percentile of the control distribution for each ACPA epitope, separately for each cohort (to account for age or storage differences between the cohorts and potential cutpoint variation). Since some of the targeted proteins had several epitopes tested, we defined elevated ACPA as having any targeted epitope within the same protein as positive. Similarly, we defined a cutpoint for the research CCP assay as >99th percentile for the control distribution within each cohort. Taken together, we established a sensitive and a specific definition of elevated ACPA in the research assay. The sensitive definition of ACPA positivity was defined as any ACPA or research CCP positivity. The specific definition of ACPA positivity was defined as two or more ACPA targeting different proteins (or one ACPA targeting a specific protein along with research CCP positivity). The research ACPA assay was performed on a total of *n* = 1128 RA cases and matched controls.

#### Commercial CCP2 assay

Stored plasma was tested for CCP autoantibodies using the second-generation Diastat enzyme-linked immunosorbent assay (Axis-Shield Diagnostics Limited, Dundee, UK). Analysis was performed at the Clinical Immunology Laboratory at Brigham and Women’s Hospital (Boston, MA, USA). CCP2 levels were reported in units/mL. At the time, the manufacturer suggested a threshold of >5 units/mL as a positive test [42]. The specific definition of CCP2 used this cutpoint to define pre-RA cases as positive at the time of blood draw. A previous study by our group using pre-RA banked blood reported that a lower threshold of >3 units/mL increased sensitivity for future RA prediction without sacrificing specificity [42]. Therefore, we defined the sensitive definition of CCP2 positivity as >3 units/mL. The commercial CCP2 assay was performed on a total of *n* = 450. Only one out of the *n* = 286 matched controls tested (0.3%) had CCP2 >5 units/mL.

#### Definitions of ACPA positivity

We used a composite of both the research ACPA and commercial CCP2 assays to define ACPA positivity for these analyses. The overall sensitive definition of ACPA positivity for pre-RA cases was defined as positivity of any research ACPA, positivity of the research CCP, or commercial CCP2 level >3 units/mL. We used this definition in our primary analysis since we expected relatively few cases to have ACPA elevation in blood banked many years prior to clinical diagnosis. For a secondary analysis, the specific definition of ACPA positivity for pre-RA cases was defined as positivity on ≥2 research ACPA proteins or CCP2 >5 units/mL, as in a previous study [38]. Since ACPA elevation is uncommon in the general population, we used these ACPA definitions to phenotype the cases only as being pre-RA ACPA+ or pre-RA ACPA−.

### Identification of asthma prior to blood donation

The primary exposure of interest was presence vs. absence of asthma at the time of blood draw. All participants were asked for research purposes whether they had been diagnosed with incident asthma by a physician, starting on the 1988 questionnaire in the NHS and the 1991 questionnaire in the NHSII. Since we hypothesized that asthma may affect ACPA in blood prior to RA diagnosis, we only considered presence or absence of asthma on questionnaires that were returned prior to blood donation. Women who self-reported asthma were sent a supplemental questionnaire to gather more detailed information on symptoms, test results, and medication use. Due to sample size limitations of this biomarker study, we relied on self-report for presence or absence of asthma. The nurses analyzed as “no asthma” in this study had reported no asthma on every research questionnaire up to the date of blood donation. A previous validation study performed in the NHS in the late 1990s (similar to when asthma was assessed in this study) found that self-report of asthma by these health professionals (registered nurses) had a positive predictive value of 81% compared to the gold standard determined by medical record review [43].

### Covariates

We considered potential confounders which were previously shown to be related to asthma [43–45] or RA [3, 46, 47]. Covariates were assessed using questionnaires before the time of blood donation. Age and race (dichotomized as white/non-white) were self-reported. Smoking pack-years were calculated using self-report of the duration and number of cigarette packs smoked to obtain a continuous value. We also categorized smoking as follows: never, >0 to 10, or >10 smoking pack-years, since smoking beyond a threshold of 10 pack-years may increase RA risk [4, 48]. As an assessment for passive smoking, women reported whether either of their parents smoked at home during their childhood and whether they ever lived with a smoker for >1 year. Continuous body mass index (BMI) was calculated using self-reported height and weight.

### Statistical analysis

We reported the proportion of pre-RA cases that had elevated ACPA by targeted protein, the research CCP assay, both commercial CCP2 cutpoints, and the composite definitions of ACPA positivity for the sensitive and specific definitions.

We reported descriptive statistics for cases based on presence or absence of elevated ACPA by the sensitive definition in pre-RA donated blood as well as their matched controls. We tested for differences between cases and their matched controls using the Wilcoxon signed-rank test for continuous variables and chi-square test for categorical variables. For RA cases, we also reported the proportion that were ACPA+ or RF+ at diagnosis from clinical testing obtained by medical record review.

We classified each RA case as pre-RA ACPA+ or pre-RA ACPA− and performed separate conditional logistic regression analyses maintaining the matched controls for each analysis. The independent variable was clinician-diagnosed asthma (yes/no) and the outcome variable was RA case status in each analysis. Odds ratios (ORs) and 95% confidence intervals (CIs) for RA case status were obtained using conditional logistic regression. We initially performed unadjusted analyses. Since controls were matched by age at blood draw, time from blood draw to index date, cohort, fasting status, menopausal status, and postmenopausal hormone use, the unadjusted analyses controlled for these variables. We then performed multivariable analyses by adjusting for continuous smoking pack-years, passive smoking, and continuous BMI. The primary analysis used the sensitive definition of elevated ACPA while the secondary analysis used the specific definition of elevated ACPA. Since we were interested in evaluating whether asthma affected the RA phenotypes (pre-RA ACPA+ or pre-RA ACPA−) differently we tested for *p* for heterogeneity of the two effect size estimates using the Wald test.

To evaluate whether asthma might also affect serostatus at time of clinical presentation, we performed conditional logistic regression analyses related to the RA phenotyping at time of clinical diagnosis using medical records considering three separate outcomes: all RA, seropositive RA, and seronegative RA. For the multivariable model, we used the same covariates that were used in the primary analysis.

We performed several sensitivity analyses. To assess for possible residual confounding from smoking, we performed a sensitivity analysis among never smokers for the outcome of pre-RA ACPA+ RA using conditional logistic regression. To further assess how smoking may affect pre-RA ACPA+ RA, we performed a cross-classified analysis of asthma (present vs. not) and smoking (dichotomized as never or ≤10 pack-years vs. >10 pack-years). In this analysis, there were four exposure categories: no asthma and ≤10 pack-years (reference group), no asthma and >10 pack-years, asthma and ≤10 pack-years, and asthma and >10 pack-years. To test for possible synergy between asthma and smoking, we tested for multiplicative interaction. Finally, since ACPA may become elevated within 5 years of clinical RA diagnosis [39, 41], we performed a sensitivity analysis restricted to the subset of cases who were diagnosed within 5 years of blood donation. Finally, we restricted the analysis to the controls to assess whether asthma was associated with ACPA+ among those who did not progress to RA.

We defined a two-sided *p* < 0.05 as statistically significant. All analyses were performed using SAS v.9.4 (Cary, NC, USA).

## Results

### Elevated pre-RA ACPA for RA cases

The proportion of RA cases with elevated research ACPA or commercial CCP2 assays (tested on blood collected prior to the index date of clinical RA diagnosis) is shown in Table 1. Among the 284 cases, 30.1% had elevated levels of any targeted protein in the research ACPA assay, with 19.5% testing positive for one or more of the anti-cit-fibrinogen epitopes. Among the cases, 30.4% had CCP2 >3 units/mL and 23.8% had CCP2 >5 units/mL. Using the sensitive definition of ACPA+, 33.8% of the cases were ACPA+, and 21.1% were ACPA+ by the specific definition.

**Table 1:** Proportion of cases (*n* = 284) with elevation on the research ACPA and commercial CCP2 assays on blood bank prior to the index date of clinical RA diagnosis in the Nurses’ Health Studies

	Proportion of pre-RA cases (*n* = 284)
Research ACPA assay results	
Elevated^a^ research ACPA by targeted protein, %	
Anti-cit-apolipoprotein A	2.8
Anti-cit-apolipoprotein E	8.5
Anti-cit-biglycan	8.5
Anti-cit-clusterin	11.7
Anti-cit-enolase	6.4
Anti-cit-fibrinogen	19.5
Anti-cit-fibronectin	7.8
Anti-cit-filaggrin	8.9
Anti-cit-histone 2A	12.8
Anti-cit-histone 2B	8.9
Anti-cit-vimentin	14.9
CCP (research)	15.6
Any research ACPA+ or CCP+ (sensitive), %	30.1
≥2 research ACPA+ or CCP+ (specific), %	20.2
Commercial CCP2 assay results	
CCP2 >3 units/mL (sensitive cutpoint), %	30.4
CCP2 >5 units/mL (clinical cutpoint), %	23.8
Definitions of ACPA positivity	
Primary/sensitive: any research ACPA+ or CCP2 >3 units/mL, %	33.8
Secondary/specific: ≥2 research ACPA+ or CCP2 >5 units/mL, %	21.1

The research ACPA assay was performed on a total of *n* = 1128 RA cases and matched controls. The commercial CCP2 assay was performed on a total of *n* = 450 RA cases and matched controls

*ACPA* anti-citrullinated protein antibodies (research test), *CCP2* second-generation cyclic citrullinated peptide (commercial test for research purposes), *RA* rheumatoid arthritis

^a^Elevated ACPA was defined as >99th percentile of the control distribution for each cohort (NHS or NHSII). Some of the targeted proteins were tested for two or more epitopes on the research ACPA assay. Separate cutpoints were determined for each batch

### Study sample

Characteristics of the study sample (*n* = 1133) at the time of blood draw are shown in Table 2 according to pre-RA ACPA status by the sensitive definition for RA cases (*n* = 96) and their matched controls (*n* = 286). For pre-RA ACPA+ cases and their matched controls, mean age was about 51 years and time from blood draw to index date was 8.1 years. For pre-RA ACPA− cases and their matched controls, mean age and time from blood draw to index date were 52 and 10.5 years, respectively.

**Table 2:** Characteristics at the time of blood draw according to pre-RA ACPA results for RA cases and their matched controls in the Nurses’ Health Studies (*n* = 1133)

	Pre-RA ACPA+ cases^b^ (*n* = 96)	Matched controls (*n* = 286)	*p* value	Pre-RA ACPA− cases (*n* = 188)	Matched controls (*n* = 563)	*p* value
Mean age, years (SD)^a^	51.3 (7.3)	50.8 (7.7)	0.67	51.6 (8.2)	51.9 (8.0)	0.65
Mean time to RA diagnosis or matched index date for controls, years (SD)^a^	8.1 (5.8)	8.1 (5.8)	0.96	10.5 (5.6)	10.5 (5.6)	0.99
CCP+ at diagnosis by medical record review, %	58.3	N/A	–	9.4	N/A	–
RF+ at diagnosis by medical record review, %	70.8	N/A	–	52.7	N/A	–
CCP+ or RF+ at diagnosis by medical record review, %	79.2	N/A	–	53.7	N/A	–
White, %	96.9	96.5	0.86	92.6	96.8	0.01
Mean body mass index, kg/m^2^ (SD)	26.1 (5.2)	24.9 (4.2)	0.05	25.6 (4.6)	25.0 (4.7)	0.08
Mean pack-years (SD)	13.3 (17.6)	11.1 (17.2)	0.18	11.1 (14.7)	8.6 (14.8)	0.02
Mean pack-years among smokers (SD)	22.4 (17.9)	21.0 (18.8)	0.45	21.4 (14.1)	19.0 (17.0)	0.02
Smoking pack-year category, %						
Never	40.6	47.2	0.24	47.9	54.7	0.005
>0 to 10	17.7	20.6		13.8	19.2	
>10	41.7	32.2		38.3	26.1	
Parent(s) smoked at home during childhood, %	68.8	66.1	0.63	62.8	63.4	0.87
Ever lived with a smoker, %	64.6	53.9	0.07	58.0	57.9	0.99
Asthma, %	17.7	6.3	0.0008	8.0	8.5	0.81

*ACPA* anti-citrullinated protein antibodies, *CCP* cyclic citrullinated peptide (clinically tested), *RA* rheumatoid arthritis, *RF* rheumatoid factor, *SD* standard deviation

^a^Age at index date and time from blood collection to index date were matching factors

^b^Pre-RA ACPA by sensitive definition: any research ACPA+ or CCP2 >3 units/mL

By the sensitive definition, 58.3% of pre-RA ACPA+ cases were CCP+ (many were diagnosed with RA prior to CCP availability), 70.8% were RF+, and 79.2% were seropositive for either according to clinical laboratory testing at time of diagnosis. Among pre-RA ACPA− cases, 9.4% were CCP+, 52.7% were RF+, and 53.7% were seropositive for either at time of clinical presentation.

Women in the pre-RA ACPA+ case group tended to have higher BMIs than their matched controls and did not show statistically significant differences in smoking. Pre-RA ACPA+ cases were more likely to report asthma at the time of blood draw than their matched controls (17.7% vs. 6.3%, *p* = 0.0008).

### Asthma and RA risk by pre-RA ACPA status

Table 3 shows the results of the primary analysis evaluating the association between asthma and RA by ACPA elevation or absence in blood using the sensitive definition. Asthma had an OR of 3.70 (95%CI 1.70,8.05) for pre-RA ACPA+ RA compared to no asthma, conditioned on matching factors. The association remained after additional adjustment in the multivariable model (OR of 3.57, 95%CI 1.58,8.04). We observed no association between asthma and pre-RA ACPA− RA. There was a significant difference in the associations between asthma and RA based on pre-RA ACPA status (*p* for heterogeneity = 0.004). The secondary analysis using the specific definition of ACPA+ showed a similar but attenuated association between asthma and pre-RA ACPA+ RA (unadjusted OR 2.75, 95%CI 1.11,6.85; multivariable OR 2.27, 95%CI 0.84,6.16; Additional file 1: Table S1).

**Table 3:** Odds ratios for incident RA by elevated or normal levels of pre-RA ACPA using conditional logistic regression in the Nurses’ Health Studies

	OR (95%CI) adjusted for matching factors^a^	Multivariable^b^ OR (95%CI)
Pre-RA ACPA+ RA *n* = 96 outcomes from total *n* = 382		
No asthma	1.00 (Ref)	1.00 (Ref)
Asthma	3.70 (1.70, 8.05)	3.57 (1.58, 8.04)
Pre-RA ACPA− RA *n* = 188 outcomes from total *n* = 751		
No asthma	1.00 (Ref)	1.00 (Ref)
Asthma	0.93 (0.50, 1.72)	0.87 (0.46, 1.61)
*p* for heterogeneity	0.006	0.004

*ACPA* anti-citrullinated protein antibodies, *CI* confidence interval, *OR* odds ratio, *RA* rheumatoid arthritis

^a^Cases and controls were matched by age at index date, time from blood draw to index date, cohort, calendar year, fasting status, menopausal status, and postmenopausal hormone use

^b^Additionally adjusted for smoking (continuous pack-years), parental passive smoking (yes/no), ever lived with smoker (yes/no), and body mass index (continuous, kg/m^2^)

### Asthma and RA risk based on serologic phenotype at time of diagnosis

Table 4 shows the results of asthma and RA risk based on serologic phenotype at time of clinical diagnosis using medical records instead of the pre-RA research blood draw. Compared to no asthma, asthma was not associated with all RA (OR 1.45, 95%CI 0.91,2.31) or seronegative RA (OR 0.97, 95%CI 0.43,2.21), but was associated with seropositive RA (OR 1.79, 95%CI 1.01,3.18).

**Table 4:** Odds ratios for incident RA, overall and by serologic phenotype determined by medical record review from clinical testing at diagnosis, using conditional logistic regression in the Nurses’ Health Studies

	OR (95%CI) adjusted for matching factors^a^	Multivariable^b^ OR (95%CI)
All RA *n* = 284 outcomes from total *n* = 1133		
No asthma	1.00 (Ref)	1.00 (Ref)
Asthma	1.55 (0.98, 2.45)	1.45 (0.91, 2.31)
Seropositive RA *n* = 177 outcomes from total *n* = 706		
No asthma	1.00 (Ref)	1.00 (Ref)
Asthma	1.94 (1.10, 3.40)	1.79 (1.01, 3.18)
Seronegative RA *n* = 107 outcomes from total *n* = 427		
No asthma	1.00 (Ref)	1.00 (Ref)
Asthma	1.00 (0.44, 2.27)	0.97 (0.43, 2.21)

*CI* confidence interval, *OR* odds ratio, *RA* rheumatoid arthritis

^a^Cases and controls were matched by age at index date, time from blood draw to index date, cohort, calendar year, fasting status, menopausal status, and postmenopausal hormone use

^b^Additionally adjusted for smoking (continuous pack-years), parental passive smoking (yes/no), ever lived with smoker (yes/no), and body mass index (continuous, kg/m^2^)

### Asthma and risk for pre-RA ACPA+ RA among never smokers

Table 5 shows the subgroup analysis for asthma and risk for pre-RA ACPA+ RA, restricting to never smokers. Compared to no asthma, asthma remained associated with pre-RA ACPA+ RA (OR 4.62, 95%CI 1.28,16.64), after conditioning on matching factors and adjusting for other potential confounders.

**Table 5:** Odds ratios for pre-RA ACPA+ RA using conditional logistic regression, restricted to never smokers (*n* = 39 outcomes from total *n* = 156) in the Nurses’ Health Studies

	OR (95%CI) adjusted for matching factors^a^	Multivariable^b^ OR (95%CI)
No asthma	1.00 (Ref)	1.00 (Ref)
Asthma	4.56 (1.32, 15.70)	4.62 (1.28, 16.64)

*CI* confidence interval, *OR* odds ratio

^a^Cases and controls were matched by age at index date, time from blood draw to index date, cohort, calendar year, fasting status, menopausal status, and postmenopausal hormone use

^b^Adjusted for parental passive smoking (yes/no), ever lived with smoker (yes/no), and body mass index (continuous, kg/m^2^)

### Asthma, smoking, and RA risk by pre-RA ACPA status

**Fig. 1:**
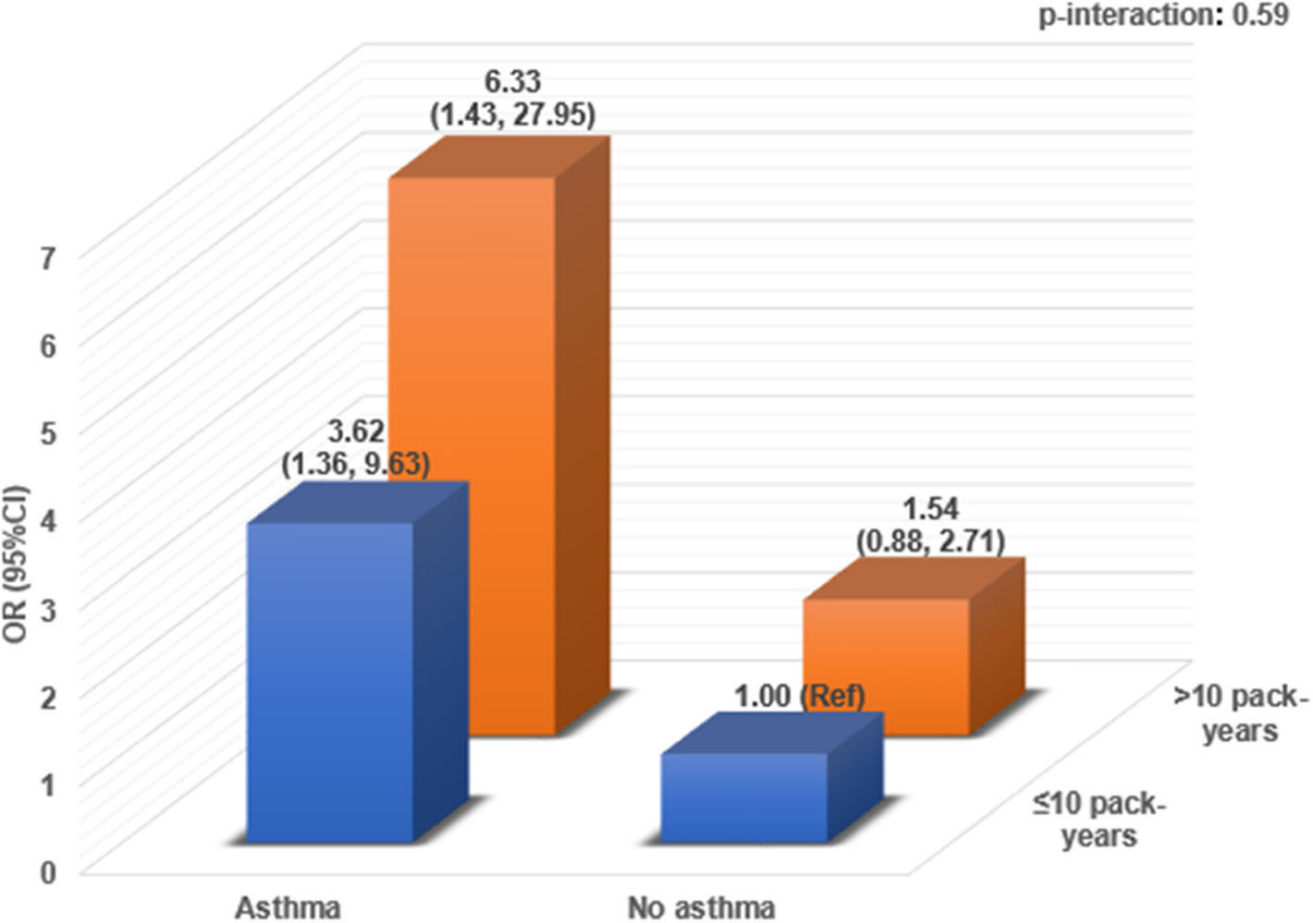
Multivariable odds ratios for pre-RA ACPA+ RA by cross-classified categories of asthma and smoking pack-years at the time of blood draw using conditional logistic regression (*n* = 96 outcomes from total *n* = 382) in the Nurses’ Health Studies. CI, confidence interval; OR, odds ratio

The analysis cross-classifying asthma and smoking for pre-RA ACPA+ RA risk is shown in Fig. 1. Asthma and >10 pack-years of smoking had a multivariable OR of 6.33 (95%CI 1.43,27.95) for pre-RA ACPA+ RA compared to the reference group of no asthma and ≤10 pack-years of smoking. There was no statistical multiplicative interaction between asthma and smoking for pre-RA ACPA+ RA risk (*p* = 0.59).

### Asthma and risk for pre-RA ACPA+ RA within 5 years of blood draw

There were 36 cases that were pre-RA ACPA+ within 5 years of RA diagnosis. When restricting to this subgroup and their matched controls, women with asthma had a multivariable OR of 4.29 (95%CI 0.95,19.77) for pre-RA ACPA+ RA compared to those without asthma (Additional file 1: Table S2).

### Asthma and ACPA+ restricted to controls

There were a total of 8 ACPA+ controls that reported asthma (11.1% of the ACPA+ controls) vs. 58 ACPA− controls that reported asthma (7.5% of the ACPA− controls, *p* = 0.26).

## Discussion

In this nested case-control study involving 1133 women, we found that clinician-diagnosed asthma was associated with increased risk for pre-RA ACPA+ RA, independent of confounders including smoking intensity/duration. This association remained strong even among individuals who never smoked. Using clinical laboratory measures at time of diagnosis, asthma was also associated with increased seropositive RA risk. Thus, asthma may be a risk factor for RA-related autoantibody development before clinical RA onset. These results provide further support for the paradigm that inflammation in the airway mucosa may contribute to the loss of immune tolerance, leading to protein citrullination, and eventual formation of ACPA preceding RA symptoms.

Previous studies have also suggested an association between asthma and RA risk, but ours is the first to focus on serostatus [24–32]. Several case-control studies established that asthma may be an RA risk factor, but had the potential for recall bias [26, 31, 32]. Since data were nested within a prospective cohort, our study has less potential for recall bias. Additionally, retrospective cohorts analyzed asthma and overall risk for RA [24, 25, 27–29]. Studies using administrative datasets also reported a relationship between asthma and RA risk, but lacked data on smoking or RA serostatus [24, 27–29]. The pre-RA ACPA+ case group in our study had similar levels of smoking compared to their matched controls. This may have been due to low sample size or related to high proportion of women with asthma among pre-RA ACPA+ cases, who may have avoided smoking due to possibly exacerbating their chronic lung disease. Despite these similar smoking levels, we showed that women with asthma had very elevated risk for pre-RA ACPA+ RA at the time of blood draw as well as risk for future seropositive RA. Our results advance these findings by incorporating detailed prospective measures on smoking and stratifying by RA serostatus at pre-RA blood draw and at clinical presentation.

Prior case-control studies have investigated the relationship between asthma and RA adjusting for smoking status and other potential confounders. For example, a Korean cross-sectional study found that asthma was associated with RA after adjusting for smoking and obesity [30] but lacked temporality and data on RA serostatus [30]. Two other case-control studies similarly found that asthma was associated with increased RA risk after adjusting for smoking and other RA risk factors [31, 32]. These studies adjusted for smoking status so may have had residual confounding from smoking intensity or duration [30–32]. Thus, our findings add to the literature by providing further evidence that asthma may be a risk factor for RA independent of smoking.

Previous studies provided the foundation for the hypothesis that pulmonary mucosal inflammation may be an initiating site for seropositive RA [49–51]. Individuals at risk for RA due to serum elevation of RA-related autoantibodies but without inflammatory arthritis were more likely to have airway abnormalities on high-resolution computed tomography scans than healthy autoantibody controls [49]. Another study showed elevated ACPA and RF levels in the sputum of unaffected first-degree relatives of patients with RA that preceded and correlated with serum elevation [50]. A recent study extended these findings by showing that ACPA elevation in sputum was associated with neutrophil extracellular traps (NETs) as a potential biologic mechanism for ACPA production in inflamed pulmonary mucosa [51]. Respiratory symptoms (such as wheezing or exercise-induced dyspnea) or diseases (such as asthma) were not investigated in these cross-sectional studies. However, airway abnormalities on imaging, elevation of ACPA in sputum, and the association of sputum RA-related autoantibodies with NETs all suggest a link between obstructive lung disease and RA-related autoantibodies prior to clinical RA onset. Patients with untreated early seropositive RA have citrullinated proteins and elevated ACPA in serum, sputum, and bronchoalveolar lavage fluid and are more likely to have lung injury and lymphoid aggregates on transbronchial biopsies than healthy controls [13, 52, 53]. Thus, our results are consistent with and complement our findings showing an association of asthma with pre-RA ACPA elevation.

Ours is the first study to investigate asthma and ACPA elevation prior to RA onset. We found a marked difference in the association of asthma on RA risk based on elevation or absence of ACPA in blood banked years prior to RA diagnosis. Women with asthma were nearly fourfold more likely to be pre-RA ACPA+ prior to RA onset. This association was also detected for seropositive RA that was determined by medical record review from clinical testing at time of RA diagnosis. These results are unlikely to be confounded by smoking as the models adjusted for both continuous pack-years and passive exposure to cigarette smoke, and the association remained strong in the non-smoker subgroup. It is also possible that subclinical pulmonary mucosal injury related to ACPA development may pre-dispose patients to clinical lung diseases at a later point in the preclinical RA phases, or after articular RA onset. Future studies should investigate this possible bi-directional association of asthma and other lung diseases with ACPA over the RA disease course. Overall, these results indicate that diseases of chronic airway inflammation may be important in RA pathogenesis, contributing to ACPA development in the years preceding clinical RA onset.

Inflammation and damage at the airway mucosa may lead to protein citrullination and other post-translational modifications which could stimulate ACPA production after antigen presentation to T cells, giving rise to local and then systemic autoimmunity [2]. Activated immune cells may initiate production of pro-inflammatory cytokines, which in turn induces protein citrullination and formation of neoantigens which are presented to T cells [12, 18]. The resulting cascade may stimulate ACPA production, initially at mucosal surfaces, such as the airways [54]. Lung tissue obtained from these patients also revealed high levels of citrullinated proteins, such as PAD2 and PAD4, suggesting the importance of the mucosal surfaces and citrullination [55]. Autophagy of citrullinated peptides occurring specifically in inflamed airways may be another biologic mechanism linking asthma to ACPA production [56, 57]. When the analysis was restricted to controls, there was a numerical increase in the proportion of those who reported asthma among the ACPA+ (11.1%) vs. ACPA− (7.5%) controls, but this did not reach statistical significance, likely due to the low number of ACPA+ controls. Given our results showing a link between asthma and pre-RA ACPA+, we hypothesize that some patients with asthma may have elevated ACPA levels in serum, induced sputum, or bronchoalveolar lavage fluid and these individuals are more likely to develop RA. The biologic mechanisms behind the association between asthma and ACPA+ RA deserve further study.

Our study has several strengths. Our study used data prospectively collected from a large cohort with many years of prospective follow-up for research purposes, which allowed for analyses utilizing banked blood, medical history, and adjustment variables that were all collected prior to RA onset. We were able to use the longitudinal data to investigate the association of asthma on ACPA development years prior to RA presentation to broaden the understanding of the timing between asthma, RA-related autoantibody development, and clinical RA onset. All RA cases in our analysis were incident after the blood donation, met established research criteria, and had clinical laboratory testing results available. Finally, we had detailed data on variables, including smoking pack-years and passive smoking, allowing us to adjust for the effect of smoking, stratify based on smoking levels, and investigate non-smokers.

Our study also has potential limitations. Asthma data were collected by questionnaire rather than formal in-person medical evaluation from health professionals whose self-report has acceptable, but not perfect, validity [43]. A subset of women who reported asthma provided more details on symptoms, test results, and medications, but we were unable to incorporate these into our analyses due to sample size limitations. We assessed for asthma up to the time of blood donation that occurred in the 1990s, so it is possible that definitions of asthma could have changed since then. However, since misclassification of the exposure typically biases toward the null, this is unlikely to explain the results showing marked increase in pre-RA ACPA+ for women who reported asthma. It is possible that some women who reported asthma and had ACPA elevation may have had bronchiectasis, typically a late extra-articular manifestation of severe RA [58–60], which was not ascertained. Since blood was drawn years prior to RA diagnosis, bronchiectasis occurring prior to RA onset would still implicate the airways as a site of ACPA production. Given the large and nationally dispersed sample, we were unable to investigate asthma subtypes/severity or duration of asthma. Future studies are needed to understand how different asthma phenotypes or their treatment may differently affect RA risk. While we were able to measure ACPA in blood banked years prior to RA, research measures on RF in pre-RA banked blood were not performed, and clinical CCP2 (with accepted cutpoints) was only tested on a subset. Further, our analysis used a composite definition of ACPA+ which may have misclassified individuals. There was only a single research blood donation tested for ACPA, so it is possible that some pre-RA ACPA− later seroconverted. We mitigated this by performing an analysis based on serostatus at diagnosis and by performing subgroup analysis on women who donated blood within 5 years of RA onset. Since the study was comprised of female nurses who were mostly healthy, white, educated, and working at baseline, the sample may not be generalizable to other populations. As in all observational studies, unmeasured confounders, such as health care utilization, may have affected our results.

## Conclusions

In summary, we showed that asthma may be important in the pathogenesis of ACPA+ RA independent of smoking. Our study provides evidence that airways may be important in RA-related autoantibody development and adds to the mucosal paradigm for RA pathogenesis. These findings suggest that individuals with asthma may be susceptible to developing RA. Further research is warranted to understand the link between pulmonary abnormalities, autoimmunity, and RA.

## Supplementary information

**Additional file 1: Table S1.** Odds ratios for RA by elevated or absent levels of pre-RA ACPA by the secondary/specific ACPA definition using conditional logistic regression in the Nurses’ Health Studies. **Table S2.** Odds ratios for pre-RA ACPA+ for RA within 5 years of RA diagnosis and their matched controls using conditional logistic regression (*n* = 36 outcomes from total *n* = 143) in the Nurses’ Health Studies.

## Data Availability

Data are available by request to the corresponding author.

## Abbreviations

ACPA: Anti-citrullinated protein antibodies (research test)
CCP2: Second-generation cyclic citrullinated peptide (commercial test for research purposes)
RA: Rheumatoid arthritis
RF: Rheumatoid factor
BMI: Body mass index
OR: Odds ratio
CI: Confidence interval
SD: Standard deviation
ACR: American College of Rheumatology
EULAR: European League Against Rheumatism
NHS: Nurses’ Health Study
