# Supplementary material for: Asthma and elevation of anti-citrullinated protein antibodies prior to the onset of rheumatoid arthritis
Source: Arthritis Res Ther. 2019 Nov 21;21:246. doi: 10.1186/s13075-019-2035-3 (PMC6868779; doi:10.1186/s13075-019-2035-3)
Supplement: Supplementary file 1 — Additional file 1: Table S1. Odds ratios for RA by elevated or absent levels of pre-RA ACPA by the secondary/specific ACPA definition using conditional logistic regression in the Nurses’ Health Studies. Table S2. Odds ratios for pre-RA ACPA+ for RA within 5 years of RA diagnosis and their matched controls using conditional logistic regression (n = 36 outcomes from total n = 143) in the Nurses’ Health Studies. [file 13075_2019_2035_MOESM1_ESM.docx]

**Asthma and elevation of anti-citrullinated protein antibodies prior to the onset of rheumatoid arthritis**

Alessandra Zaccardelli, BS^1^*

Xinyi Liu, MS^1^*

Julia A. Ford, MD^1,2^

Jing Cui, MD, PhD^1,2^

Bing Lu, MD, DrPH^1,2^

Su H. Chu, PhD, MS^1,2^

Peter H. Schur, MD^1,2^

Cameron B. Speyer, BA^1^

Karen H. Costenbader, MD, MPH^1,2^

William H. Robinson, MD, PhD^3,4^

Jeremy Sokolove, MD^3,4,5^
Elizabeth W. Karlson, MD, MS^1,2^

Carlos A. Camargo, Jr., MD, DrPH^1,2,6^

Jeffrey A. Sparks, MD, MMSc^1,2^

*Contributed equally
 ^1^Brigham and Women’s Hospital, Boston, MA, USA
^2^Harvard Medical School, Boston, MA, USA
^3^Stanford University School of Medicine, Palo Alto, CA, USA
^4^VA Palo Alto Health Care System, Palo Alto, CA, USA
^5^Abbvie, Redwood City, CA, USA
^6^Massachusetts General Hospital, Boston, MA, USA

**Correspondence and reprint requests:**

Jeffrey A. Sparks, MD, MMSc
Division of Rheumatology, Immunology and Allergy
Brigham and Women’s Hospital
60 Fenwood Road, #6016U
Boston, MA 02115
Phone: (617) 525-1040
Fax: (617) 732-5766
Email: [jsparks@bwh.harvard.edu](mailto:jsparks@bwh.harvard.edu)

**Table S1**. Odds ratios for RA by elevated or absent serum levels of pre-RA ACPA by the secondary/specific ACPA definition using conditional logistic regression in the Nurses’ Health Studies.

|  | **OR (95%CI) adjusted for matching factors*** | **Multivariable** OR (95%CI)** |
| --- | --- | --- |
| ***Pre-RA ACPA+ RA***  *n=60 outcomes from total n=239* |  |  |
| No asthma | 1.00 (Ref) | 1.00 (Ref) |
| Asthma | 2.75 (1.11, 6.85) | 2.27 (0.84, 6.16) |
| ***Pre-RA ACPA- RA*** *n=224 outcomes from total n=894* |  |  |
| No asthma | 1.00 (Ref) | 1.00 (Ref) |
| Asthma | 1.27 (0.74, 2.19) | 1.22 (0.71, 2.10) |
|  |  |  |
| *p* for heterogeneity | 0.15 | 0.15 |

ACPA, anti-citrullinated protein antibodies; CI, confidence interval; OR, odds ratio; RA, rheumatoid arthritis.

*Cases and controls were matched by age at index date, time from blood draw to index date, cohort, calendar year, fasting status, menopausal status, and postmenopausal hormone use.
**Additionally adjusted for smoking (continuous pack-years), parental passive smoking (yes/no), ever lived with smoker (yes/no), and body mass index (continuous, kg/m^2^).

**Table S2**. Odds ratios for pre-RA ACPA+ for RA within 5 years of RA diagnosis and their matched controls using conditional logistic regression (n=36 outcomes from total n=143) in the Nurses’ Health Studies.

|  | **OR (95%CI) adjusted for matching factors*** | **Multivariable** OR (95%CI)** |
| --- | --- | --- |
| No asthma | 1.00 (Ref) | 1.00 (Ref) |
| Asthma | 5.60 (1.40, 22.38) | 4.29 (0.93, 19.77) |

ACPA, anti-citrullinated protein antibodies; CI, confidence interval; OR, odds ratio; RA, rheumatoid arthritis.

*Cases and controls were matched by age at index date, time from blood draw to index date, cohort, calendar year, fasting status, menopausal status, and postmenopausal hormone use.
*Adjusted for smoking (continuous pack-years), parental passive smoking (yes/no), ever lived with smoker (yes/no), and body mass index (continuous, kg/m^2^)
